# RNA Interference to Suppress Flaviviral Encephalitis

**DOI:** 10.1371/journal.pmed.0030139

**Published:** 2006-02-14

Mosquito-borne flaviviruses such as Japanese encephalitis virus (JEV) and West Nile virus (WNV) are two of the most important examples of emerging and resurging pathogens. Currently, there are no effective drugs available to treat these infections. Moreover, infections by diverse neurotropic flaviviruses are clinically indistinguishable, which makes it important to develop broad-based therapies effective against multiple flaviviruses.

In a new study, Priti Kumar and colleagues examined whether RNA interference (RNAi)–based intervention could suppress lethal JEV and WNV encephalitis in mice. RNAi is a process in which short double-stranded RNA (dsRNA) called short interfering RNA (siRNA) inhibits gene expression in a sequence-specific manner. This process was first discovered in plant cells, and there is still speculation concerning its implications for human physiology. However, RNAi has emerged as a powerful technique for posttranscriptional gene silencing, useful in both research and, potentially, the development of new therapies.

Studies of RNAi in plants, invertebrates, and, more recently, mammalian cells have all hinted at the potential of this method. RNAi-mediated posttranscriptional gene silencing appears to have evolved to protect against invading genetic elements such as transposons and viruses. In mammals, although exposure to dsRNAs greater than 30 base pairs in length induces an antiviral interferon response that represses mRNA translation globally, shorter siRNA introduced into mammalian cells leads to specific mRNA degradation without activating the interferon response. 

Previous work has shown that infection by respiratory syncytial virus (RSV) and parainfluenza virus (PIV) could be specifically prevented and inhibited by siRNAs. In an RSV mouse model, researchers used siRNA to target the P protein, a key subunit of the viral RNA–dependent RNA polymerase, and found that it strongly inhibited RSV gene expression and RSV growth in culture. They also showed that single and concurrent infections in mice could be prevented and treated by specific siRNA applied exclusively intranasally. Their findings suggested that inhaled siRNA could be a promising strategy for anti-RSV and anti-PIV therapy in humans.

Studies have also demonstrated that siRNAs expressed by a lentivirus vector could prevent and treat influenza A virus (IAV) pneumonia in mice. When siRNAs specific for conserved regions of the IAV genes (nucleoprotein, acid polymerase, and basic polymerase 1) were administered prior or subsequent to the virus challenge, there were reductions in lung virus titers, lethality, or both. Based on these results, in a recent review of siRNAs for treating influenza, Jack Bennink and Tara Palmore have suggested that siRNAs could lead to a therapeutic solution against the variability of the IAV hemagglutinin. 

Kumar and colleagues from Harvard University describe promising results for the therapeutic potential of RNAi in treating viral encephalitis, both virus-specific and across species. They induced RNAi in mice, with either a lentivirally expressed short hairpin RNA or a synthetic siRNA. By targeting a species-specific sequence in cd loop coding region in domain II of the viral envelope protein for JEV or WNV, the team achieved specific protection in mice against the corresponding virus. And in addition, by targeting a sequence within the cd loop that is conserved across both viral species, they were able to protect mice against encephalitis induced by both viruses.

These results suggested that a single treatment with siRNA may be sufficient for protection against fatal encephalitis, which is encouraging from a therapeutic angle. In one set of experiments, they showed that single administration of siRNA could provide more than 60% protection even when administered 18 hours after infection. This time frame is relevant because the burst phase for JEV and WNV replication is around 18 hours—when many virus progeny are released.

However, although a single lipid–based siRNA delivery in the brain parenchyma caused some degree of lateral spread and offered protection even in an established infection, this approach is unlikely to work when the infection has spread across the brain. This observation is important for potential clinical applications since usually treatments are not given until after the appearance of clinical symptoms.

To address this limitation, the authors suggest, further studies will have to focus on developing improved methods for siRNA delivery to brain cells. And much work will need to be done to establish the safety of this approach in humans. But RNAi technology may be shifting from a laboratory tool to a realistic treatment for viral infection.

## 

**Figure:**
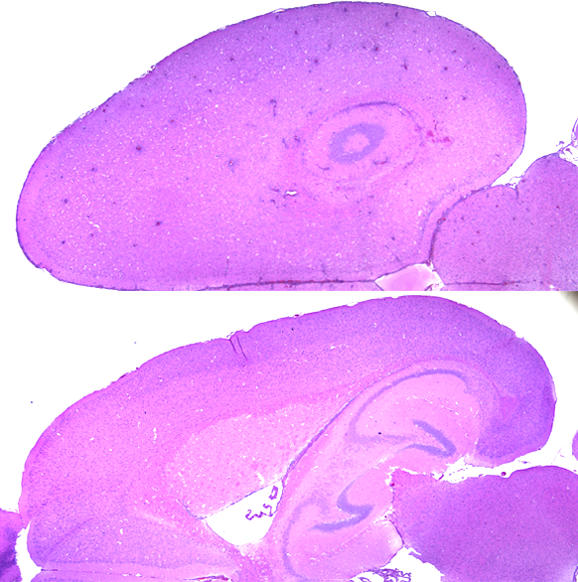
Section from JEV-infected mouse brain obtained after treatment with control or JEV-specific siRNA

